# Electrical impedance myography as a marker of muscle mass in rats with simulated Anorexia Nervosa

**DOI:** 10.2478/joeb-2025-0014

**Published:** 2025-08-06

**Authors:** Megan E. Rosa-Caldwell, Buket Sonbas Cobb, Lauren Breithaupt, Seward B. Rutkove

**Affiliations:** Energy Availability and Muscle Metabolism Laboratory, Exercise Science Research Center, Department of Health, Human Performance, and Recreation, University of Arkansas, Fayetteville, AR 72701; Beth Israel Deaconess Medical Center and Harvard Medical School, Department of Neurology, Boston, MA 02215; Department of Electrical and Electronic Engineering, Harran University, Sanliurfa, Turkey; Department of Psychiatry, Harvard Medical School, Eating Disorders Clinical and Research Program, Massachusetts General Hospital, Boston, MA 02114; Mass General Brigham Multidisciplinary Eating Disorders Research Collaborative, Mass General Brigham, Boston, MA 02114

**Keywords:** gastrocnemius, muscle assessment, eating disorders, Electrical Impedance Myography, EIM

## Abstract

**Motivation:**

Anorexia Nervosa (AN) is characterized by a severe reduction in caloric intake resulting in substantial weight loss. Methods to evaluate muscle loss specifically during AN or following a weight recovery intervention are difficult to administer and expensive.

**Purpose:**

To evaluate the utility of electrical impedance myography (EIM) to assess changes to muscle mass during simulated AN and different durations of weight recovery in rats.

**Methods:**

Female Sprague-Dawley rats (n=11/group, total of 66 rats, 8 weeks old) were divided into simulated AN or healthy control conditions. Simulated AN included 30 days of 50–60% food restriction. Following AN intervention, rats were further subdivided into recovery cohorts which included five, fifteen, or thirty days of ab libitum food consumption to elicit weight gain. EIM was assessed at various stages of weight loss and recovery and correlated to metrics of muscle mass.

**Results:**

Various EIM parameters detected changes in muscle mass both during simulated AN and following weight restoration. The resistance parameter produced the most consistent results during simulated AN and following various stages of weight recovery. Moreover, the resistance parameter had the highest correlation with gastrocnemius mass (r = ~0.50, p<0.05). Maximal tetanic plantar flexion was also analyzed but did not correlate with any EIM parameters.

**Conclusion:**

EIM can non-invasively detect changes to muscle mass during AN and following various states of weight recovery.

## Introduction

Anorexia Nervosa (AN) is a prevalent psychiatric disorder with high morbidity. Approximately 4% of women have active AN with emerging evidence suggesting the modal age of onset for AN is decreasing [1,2,3]. While AN is associated with a plethora of physiological consequences, a profound and sustained loss of skeletal muscle health is a noteworthy consequence of the condition. Our team’s recent systematic review noted a ~24.5% lower muscle size and ~35% lower muscle strength in those with AN [4] compared to individuals without AN. Additionally, in those who achieve short-term weight restoration, muscle mass does not appear to normalize [4]. This is true in both human and rodent models of AN [4,5]. Given skeletal muscle’s profound influence on mobility, whole body metabolism, and overall quality of life, this sustained muscle loss has clear implications for the short and long-term health of those with AN or a history of AN. Correspondingly, understanding how AN affects skeletal muscle size and strength during AN and the weight recovery process has clear clinical value. However, assessment of skeletal muscle size or strength is notoriously difficult.

Technologies to assess muscle size typically include MRI or CT, which can provide a measure of muscle volume, both of which are inconvenient and costly, including the associated required image analysis [6]. Dual X-ray absorptiometry (DXA) can provide a broader assessment of lean mass but cannot assess individual muscles. Assessments for muscle strength such as 1-repetition maximal strength (1-RM) testing, suffer similar downsides, mainly being inconvenient and expensive. In particular, 1-RM testing is highly reliant on trained personnel, appropriate weightlifting equipment, as well as a maximal effort of the individual being evaluated. Therefore, an evaluation tool that can assess muscle size and/or strength quickly, non-invasively, and is not reliant on participant motivation would be highly valuable.

Electrical impedance myography (EIM) is a technique developed to non-invasively and inexpensively assess disorders that impact muscle quality [7]. In brief, the technique involves applying low-intensity electrical current at multiple frequencies to a muscle across two surface electrodes while another set of surface electrodes measures the corresponding voltages [7]. The EIM electrodes are placed in relation to the specific target muscle of interest (gastrocnemius or quadricep for example). Our previous work has demonstrated EIM is capable of assessing muscle size (mass, fiber cross-sectional area, etc.) across a variety of muscle pathologies and a variety of organisms (including human, rodent, zebrafish) [8,9,10,11,12]. Moreover, EIM is sufficiently sensitive to capture longitudinal changes in muscle mass [13], implying the technique may be used as an assessment tool to monitor changes in muscle health. Hitherto, EIM has never been applied to skeletal muscle undergoing AN-induced muscle atrophy or recovery but may be a valuable tool in this specific population. With our understanding of EIM’s sensitivity to non-invasively assess muscle mass *in vivo*, the purpose of this study was to evaluate the capacity of EIM to track changes in muscle health during the development of low-weight status AN followed by recovery in an animal AN model. We hypothesized that EIM values will correlate to muscle health parameters during AN and with subsequent restoration of mass and function.

## Materials and methods

### Animal experiments

Phenotypic data for some of these animals has previously been published [5]. All rats were kept in humidity and temperature (~23° C) controlled housing on a 12:12 hour light:dark cycle. Female Sprague Dawley rats were used for this study. At eight weeks of age, rats began simulated AN. The procedure for simulating AN in these rats has been previously published [5]. Briefly, rats are kept group-housed and briefly moved to individual housing during feeding early in the morning in which rats are provided ~40–50% of *ad libitum* food consumption. Rats are given up to 120 min to consume food, after which they are returned to group housing until the following morning. This procedure is repeated for 30 days, resulting in ~20% weight loss and ~30−40% difference in weight between AN rats and healthy controls [5]. For this experiment, we had multiple study cohorts. In the first cohort, we longitudinally evaluated simulated AN compared to healthy controls (CON). In the second cohort, we evaluated rats that had undergone simulated AN for 30 days and then were provided *ad libitum* food consumption to elicit weight recovery (AN+30R). The 30-day period of recovery was chosen to match the 30-day period of simulated AN. AN-R rats were compared to age-matched healthy controls (CON+30R). Finally, in our 3^rd^ cohort, we cross-sectionally assessed rats that had undergone simulated AN, healthy controls (CON), and rats exposed to different durations (5 and 15 days) of weight recovery (AN+5R and AN+15R respectively). Following designated interventions, rats were anesthetized with 2–3% inhaled isoflurane and gastrocnemius muscles weighed and frozen in liquid nitrogen. Rats were then euthanized after muscle collection while under anesthesia by cardiac puncture.

### Maximally stimulated plantar flexion

Nerve stimulated maximal plantarflexion were completed as we have previously described using Aurora Scientific nerve stimulation apparatus with bi-phasic stimulator and dual-mode muscle lever system [14,15,16]. Briefly, rats were anesthetized with 2–3% isoflurane and remained under anesthesia for the duration of the testing (<10 min). Needles were placed ~2mm apart in the posterior portion of the leg under the knee to elicit maximal plantar flexion. To ensure appropriate needle placement, a small (10 Hz) stimulation was provided to induce a twitch. Following appropriate needle placement, muscles were provided a 200 Hz, 200 ms stimulation, eliciting maximal tetanus. Tetanus curves were evaluated for area under the curve (AUC) and are represented as mN×sec. Maximally stimulated plantar flexion was measured prior to simulated AN and after designated intervention.

### Collection of EIM data

EIM data was collected as we have previously described [11,13,17]. Briefly, rats were anesthetized with 2−3% isoflurane and the rat’s left leg was shaved. Then, the rat was placed in a prone position and the rat’s left leg was positioned with the gastrocnemius facing upwards and at ~45° angle from the rest of the body. The rat’s foot was gently taped down to ensure no additional movement of the leg, which could cause artifacts in EIM spectra. Then, an EIM 4-needle electrode array (4 mm wide array, (3 mm deep, 1 mm of distal tip exposed and inserted into the leg) was inserted into the gastrocnemius of the rat parallel to the long axis of the muscle. The EIM array was inserted in the belly of the gastrocnemius, approximately 2−3 mm from the popliteal fossa. Care was taken to ensure all needles were inserted to equal depths into the muscle. EIM was then collected at 41 predesignated frequencies ranging from 1 kHz to 10 MHz using the mView System (Myolex, Inc., Boston, MA, United States). We note that while EIM is easy to perform with surface approaches in humans, in rats, this needle-based approach can be performed more easily, there being no need to depilate the skin to remove all fur.

### Statistical analysis

All data were assessed for normality and equality of variance prior to analysis whereby we found no evidence of against normality in any of our groups (Shapiro-Wilk tests >0.05) or evidence of unequal variance between groups (Levene’s test >0.05). Initial phenotypic stats were completed in SAS (SAS^®^ Studio Release: 3.81, SAS Institute Inc. Cary, NC), for CON v. AN and CON-30R v. AN-30R. Data were analyzed by t-test with a covariate of baseline bodyweight or baseline measurement (in the case of maximal plantar flexion).

For cross-sectional analysis of CON, AN, AN+5R, and AN+15R a 1-way ANOVA was utilized with a Tukey post-hoc adjusted p-value. For all tests, significance was denoted at p<0.05. The preliminary EIM analysis focused on evaluating standard impedance data, including phase, resistance, and reactance, measured across 41 frequencies. With visual inspection of EIM spectra across phase, resistance, and reactance parameters, a noticeable pattern of extreme outliers emerged at the higher frequency range in accordance with previous works demonstrating the presence of ‘hook artifacts’ due to unintended capacitive leakage within the physical structure of the material being measured (in this case, skeletal muscle) [18]. To address this, a mean and standard error around the mean (SEM) based outlier removal algorithm was implemented, whereby if a spectrum demonstrated > 2 standard errors it was removed.

If an outlier was detected in the high-frequency range of a dataset, the entire impedance profile across all 41 frequencies was removed. This process resulted in the exclusion of 10 out of 170 datasets, corresponding to a data loss of 5.8%. There were no meaningful differences across groups or timepoints in the number of outliers. Specific data counts after outlier removal are depicted in supplementary materials.

Various EIM parameters were assessed for their capacity to correlate with gastrocnemius mass across multiple experiments. Statistical analyses for all data including EIM parameters (Fig 2–5) were performed using Python version 3.12.4, with the Scipy library (version 1.13.1) for computational support, ensuring the accuracy and reliability of the results. All data are depicted as Mean ± SEM.

All raw data are available on our Open Science Framework page for this project and can be accessed at: https://doi.org/10.17605/OSF.IO/6PVD8

### Ethical approval

The research related to animals use has been complied with all the relevant national regulations and institutional policies for the care and use of animals. All experiments were approved by the Beth Israel Deaconess Medical Center Institutional Animal Care and Use Committee (AUP 009-2022).

## Results

### Finding 1: Simulated AN results in profound changes to bodyweight, gastrocnemius mass, and muscle force production, which remain following long-term recovery

**Figure 1:**
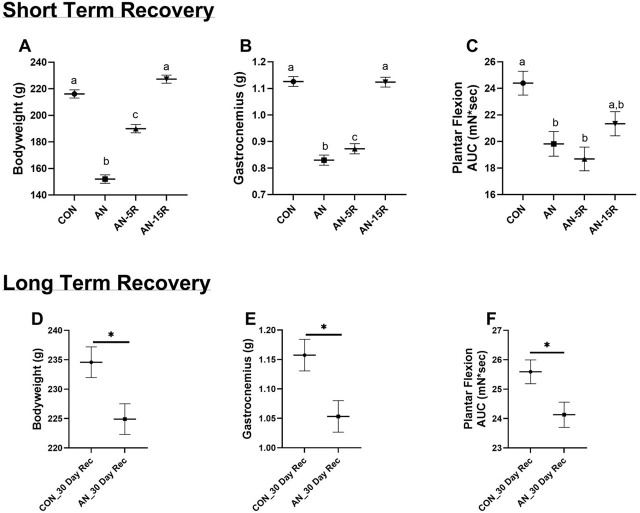
Phenotypic data from short term recovery and long-term recovery following simulated anorexia nervosa (AN) and recovery interventions of five days (AN-5R) and fifteen days (AN-15R) compared to healthy controls (CON). **A)** Bodyweight following short-term recovery, **B)** Gastrocnemius weight following short-term recovery, **C)** Plantar flexion area under the curve (AUC) of maximal tetanus following short-term recovery. **D)** Bodyweight following long-term recovery from simulated AN (AN_30 Day Rec) and age-matched controls (CON_30 Day Rec) **E)** Gastrocnemius weight following long-term recovery, **F)** Plantar flexion area under the curve (AUC) of maximal tetanus following long-term recovery. Data are presented as Mean ± SEM, different letters represent statistically different means at p<0.05, * p<0.05, n=10–11/group.

When including baseline bodyweight as a covariate, AN resulted in ~30% lower bodyweight compared to CON (p<0.001, Figure 1A). (Please note that bodyweights for some of these same animals have previously been published [5]. Bodyweight remained ~12% lower in AN+5R compared to CON (p<0.001, Figure 1A) and was no longer different from CON in AN+15R (p=0.073, Figure 1A). Similarly, as concerns gastrocnemius mass, the AN cohort was ~26% lower compared to CON (p<0.001), remained ~22% lower in AN+5R compared to CON, and was no longer different from CON in AN+15R (p=0.998, Figure 1B). Finally, the integration of maximal plantar flexion tetanus area (plantar flexion area under the curve, AUC), was ~19% lower in AN compared to CON (p=0.006), continued to deteriorate to ~23% lower in AN+5R compared to CON (p<0.001), but was no longer different from CON in AN+15R (p=0.107, Figure 1C). Importantly, CON rats were euthanized along with our no-recovery AN rats, and therefore AN+5R and AN+15R cohorts were being compared to animals that were 5−15 days younger and not to true age-matched animals.

Amongst our long-term recovery cohort, in which AN+30R were age-matched to CON+30R, when taking into account baseline bodyweight, AN+30R rats were ~4% lighter compared to CON+30R (p=0.018, Figure 1D). AN+30R rats also had ~9% lower gastrocnemius mass compared to CON+30R (p=0.014, Figure 1E) as well as ~6% lower plantar flexion AUC in AN+30R compared to CON+30R (p=0.027, Figure 1F).

### Finding 2: EIM parameters corresponded to phenotypic changes in muscle during longitudinal assessment of AN v. CON animals

We began with evaluating how well-established EIM single-frequency impedance values changed during the development of simulated AN in our acute cohort. Specifically, we assessed impedance values at 50 kHz, previously shown to be valuable and reliable for assessing muscle health in various EIM studies [19,20].

**Figure 2:**
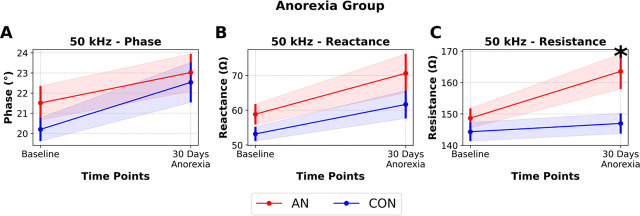
Longitudinal Electrical Impedance Myography (EIM) data at 50Hz in rats undergoing simulated anorexia nervosa (AN) or healthy controls (CON). **A)** Phase, **B)** Reactance, **C)** Resistance. Data are presented as Mean ± SEM, * p<0.05, n=10–11/group.

Indeed, consistent with prior research, we found no difference in 50 kHz phase and reactance values between AN or CON (p=0.737 & p=0.224, Figure 2A&B) but did find differences between AN and CON in 50 kHz resistance. Specifically, AN and CON had similar resistance values prior to intervention, but by the end of the intervention, AN had ~11.28% % greater resistance compared to CON (p=0.022, Figure 2C).

### EIM detects longitudinal changes in muscle loss and muscle restoration in our rodent model of AN and recovery

**Figure 3:**
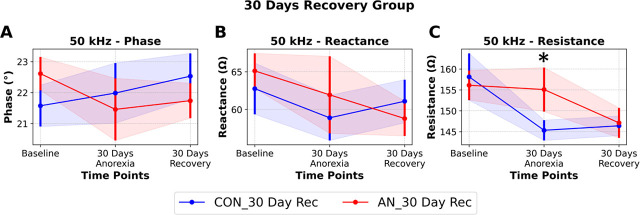
Longitudinal Electrical Impedance Myography (EIM) data at 50Hz in rats undergoing simulated anorexia nervosa followed by a recovery intervention to restore bodyweight (AN_30 Day Rec) or healthy age-matched controls (CON_30 Day Rec). **A)** Phase, **B)** Reactance, **C)** Resistance. Data are presented as Mean ± SEM, * p<0.05, n=10–11/group.

We next evaluated if EIM could detect changes in muscle loss and muscle restoration in our rodent model of AN and subsequent recovery. We did not see any differences between groups on EIM phase or reactance at 50 kHz (Figure 3A & 3B). However, similar to our results in experiment 1, we found resistance at 50 kHz to respond to changes in muscle size during muscle loss and recovery (Figure 3C), with no differences between groups at baseline (p=0.775), 6.71% difference between groups following 30 days simulated AN which approached statistical significance (p=0.10), and no difference between groups following recovery intervention (p=0.844).

### Finding 3: EIM can detect cross-sectional differences at various stages of weight recovery following AN

**Figure 4:**
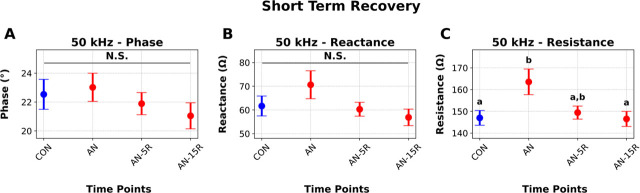
Cross-sectional Electrical Impedance Myography (EIM) data at 50Hz in rats undergoing simulated anorexia nervosa (AN), healthy controls (CON) or rats undergoing five days (AN-5R) or fifteen days (AN-15R) of recovery. **A)** Phase, **B)** Reactance, **C)** Resistance. Data are presented as Mean ± SEM, different letters represent statistical differences at p<0.05, n=10–11/group.

We examined if EIM parameters could detect cross-sectional differences across different duration of weight and muscle restoration. We note no differences between groups on 50 kHz phase or reactance (Figures 4A and 4B). However, as depicted in Figure 4C, resistance values at 50 kHz appeared to detect alterations in muscle size across timepoints (AN v. CON, p=0.031, AN v. AN+15R, p=0.025, Figure 4C).

### Finding 4: EIM parameters moderately correlate with gastrocnemius mass

**Figure 5:**
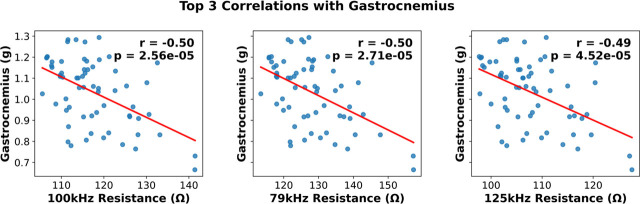
Top three correlations between Electrical Impedance Myography (EIM) parameters and gastrocnemius mass. **A)** 100 kHz Resistance, **B)** 79 kHz Resistance, and **C)** 125 kHz Resistance, n=10–11/group.

In our final analysis, we combined EIM data from all groups at their final timepoint prior to euthanasia and evaluated their correlational strength with metrics of muscle health. This exploratory analysis included correlations of all frequencies for all EIM parameters (phase, resistance, reactance). Overall, no EIM parameters significantly correlated with metrics of muscle strength such as plantar flexion AUC (top three correlation coefficients = ~0.17, p-value range = 0.17−0.18). However, EIM parameters of 100 kHz resistance, 79 kHz resistance, 125 kHz resistance resulted in moderate negative correlations with gastrocnemius size (top three correlation coefficients = −0.5, −0.5, −0.49 and associated p-values < 0.001). The top three correlations are depicted in Figures 5A–C.

## Discussion

In this study, we found that EIM is sensitive enough to detect overall changes in muscle health during AN and simulated recovery. Overall, based on these results in rats, EIM may be a useful tool for broad evaluation of muscle health in this population, but more refinement of the technique may be needed to identify subtler differences in muscle mass.

We evaluated how EIM parameters were altered (or not) across the progression and recovery of AN using well-established EIM parameters all obtained at 50 kHz. Specifically, the 50 kHz resistance parameter detected longitudinal loss of muscle mass in AN v. CON (Fig 2), longitudinal loss and gain of muscle mass in AN-R v. CON-R (Fig 3), and cross-sectional differences in muscle mass across shorter recovery periods (Fig 4). These data align with prior work in clinical and pre-clinical literature demonstrating the resistance parameter largely correlates with muscle size (both mass and volume) across a variety of conditions [10,11,12,21,22,23]. Given the simplicity of evaluating a tissue’s resistance with an EIM device, these data may suggest EIM, as applied to humans, is a non-invasive and feasible modality to quickly assess muscle mass in this population. Specifically, short-term increases in resistance of muscle may be indicative of atrophy. This parameter may allow for detection of AN or energy insufficiency without reliance on psychological assessments which can be highly unreliable. Moreover, because EIM resistance at 50 kHz changed during recovery periods, it may also have utility for evaluation of recovery trajectories and broad muscle restoration in this population.

We also evaluated how EIM parameters directly correlated with muscle mass and muscle function. Overall, we find the strongest correlations at r = ~0.50 for gastrocnemius mass. This correlation does largely agree with other clinical assessments that compare EIM to clinical outcomes such as Duchenne’s Muscular Dystrophy [12,24] as well as others [25,26,27] but is weaker than some other reports of EIM and metrics of muscle function [10,11,13]. Shorter term recovery timepoints (AN+5R) may be attenuating the strength of relationship between EIM and muscle mass whereby there were changes in the resistance EIM parameter (Fig 4), but gastrocnemius size had not changed much compared to AN (Fig 1). As noted previously, these findings suggest EIM may not be sufficient to detect subtle changes in muscle mass induced by caloric restriction. However, given the large effects of AN on overall muscle mass, EIM may still have utility for quickly and non-invasively evaluating local muscle mass in clinical populations. We also note very weak correlations between EIM parameters and metrics of muscle function, such as maximal plantar flexion AUC (r ~0.17). This could be related to the fact that EIM is only assessing broad compositional and structural aspects of the muscle and not its metabolic or physiological status. Regardless, these findings imply EIM would not be adequate to assess muscle strength changes in this population and other clinical assessments (such as grip strength) may be necessary to evaluate longitudinal changes in muscle strength.

Mechanistic differences in the etiology of other atrophic diseases such as muscular dystrophy, simulated spaceflight, aging, amyotrophic lateral sclerosis (ALS) and others may account for some of the differences we see in different EIM parameters between this study and other studies. For example, neurodegenerative diseases such as ALS or Charcot-Marie-Tooth type 1A, have muscle loss secondary to primary neuronal dysfunction [10,28]. Other more complex conditions such as muscle dystrophy or aging have many simultaneous intramuscular alterations concurrent with disease progression above and beyond simple atrophy such as fat infiltration, excessive fibrosis, and inflammation [29,30]. Finally, spaceflight-induced atrophy is also associated with fluid shift due to altered gravitational load, which also occurs in terrestrial models of spaceflight such as hindlimb unloading or partial weight-bearing [31,32]. In aggregate, differences between etiology of muscle loss and subsequent effects on the muscle (infiltration of non-contractile tissue, inflammation, fluid shift) all can contribute to differences in EIM spectra in the phase, reactance, and/or resistance parameters. Correspondingly, the potential value and application of EIM as a diagnostic or therapy-guiding tool needs to be considered separately for each neuromuscular condition.

There are a few limitations that should be acknowledged for this particular investigation. First, this study was completed in a calorie restriction-based rodent model of AN, and we cannot rule out the possibility that the psychological component of voluntary food restriction inherent to AN may have altered muscle physical properties (and correspondingly EIM parameters) that we cannot anticipate. Additionally, for this study we utilized needle-based EIM, which is very common in rodent studies leveraging this technology; however, surface EIM, where the device only sits on the skin and electrodes do not penetrate the skin, is much more commonly used and preferred in clinical settings. Correspondingly, future investigations would need to validate our current findings using surface EIM. We do note that one previous study showed a strong relationship between surface and *ex vivo* muscle EIM assessment [33]. Additionally, only female rats were used in this study due to AN affecting disproportionally more females compared to males at a ratio of ~4−8:1 [12,33]; therefore, these results would need to be further validated in male organisms. Finally, as noted previously, we only detected large differences between groups on metrics related to muscle mass. Therefore, EIM in AN may not be appropriate in instances where subtle evaluation of muscle health is necessary. Regardless of these limitations, these data are key for the future development of EIM technology in those with AN.

In conclusion, we find EIM resistance is sensitive to detect longitudinal changes in muscle mass during muscle loss and following muscle regrowth in a rat AN model. There is currently a lack of clinical guidance on management of muscle health in this population. Having a readily available diagnostic tool that can quickly and non-invasively estimate changes in muscle mass may be the first step toward developing a framework for the clinical management of muscle health in those with AN and to assist with their recovery.
